# Nanoformulation of Polyphenol Curcumin Enhances Cisplatin-Induced Apoptosis in Drug-Resistant MDA-MB-231 Breast Cancer Cells

**DOI:** 10.3390/molecules27092917

**Published:** 2022-05-03

**Authors:** Parastoo Karami, Goran Othman, Zjwan Housein, Abbas Salihi, Mohammad Ali Hosseinpour Feizi, Hewa Jalal Azeez, Esmaeil Babaei

**Affiliations:** 1 Department of Biology, School of Natural Sciences, University of Tabriz, Tabriz P.O. Box 5166616471, Iran; karamiparastoo431@yahoo.com (P.K.); pourfeizi@tabrizu.ac.ir (M.A.H.F.); hewa.azeez.ha@gmail.com (H.J.A.); 2 Department of Medical Laboratory Technology, Erbil Health and Medical Technical College, Erbil Polytechnic University, Erbil 44001, Iraq; goran.othman@epu.edu.iq (G.O.); zjwan.h@epu.edu.iq (Z.H.); 3 Department of Medical Laboratory Technology, Al-Qalam University College, Kirkuk 36001, Iraq; 4 Department of Biology, College of Science, Salahaddin University-Erbil, Erbil 44001, Iraq; abbas.salihi@su.edu.krd; 5 Center of Research and Strategic Studies, Lebanese French University, Erbil 44001, Iraq

**Keywords:** polyphenolic curcumin, cisplatin, combination therapy, breast cancer, apoptosis, gemini surfactant nanoparticles

## Abstract

Triple Negative Breast Cancer (TNBC) is the aggressive and lethal type of breast malignancy that develops resistance to current therapies. Combination therapy has proven to be an effective strategy on TNBC. We aimed to study whether the nano-formulation of polyphenolic curcumin (Gemini-Cur) would affect the cisplatin-induced toxicity in MDA-MB-231 breast cancer cells. MDA-MB-231 cells were treated with Gemini-Cur, cisplatin and combination of Gemini-Cur/Cisplatin in a time- and dose-dependent manner. Cell viability was studied by using MTT, fluorescence microscopy and cell cycle assays. The mode of death was also determined by Hoechst staining and annexin V-FITC. Real-time PCR and western blotting were employed to detect the expression of BAX and BCL-2 genes. Our data demonstrated that Gemini-Cur significantly sensitizes cancer cells to cisplatin (combination index ≤ 1) and decreases IC50 values in comparison with Gemini-cur or cisplatin. Further studies confirmed that Gemini-Cur/Cisplatin suppresses cancer cell growth through induction of apoptosis (*p* < 0.001). In conclusion, the data confirm the synergistic effect of polyphenolic curcumin on cisplatin toxicity and provide attractive strategy to attain its apoptotic effect on TNBC.

## 1. Introduction

The International Agency for Research on Cancer (IARC) reported that in 2020, an estimated 19.3 million new cancer cases and 10.0 million cancer deaths has occurred. Breast cancer is the most common malignancy in women with nearly 2.3 million new cases (24.2% of all tumor malignancies in women) diagnosed in 2020 [1]. Despite recent advances in treatment, side effects and the development of drug resistance limit the usefulness of current therapies for triple negative breast cancer [2,3].

**Figure 1:**
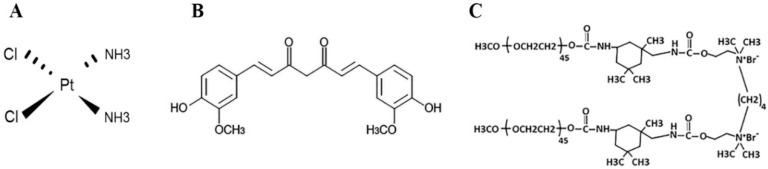
The molecular structure of (**A**) Cisplatin (**B**) Curcumin and (**C**) mPEG urethane gemini surfactant nanoparticle.

One of the classical drugs used as chemotherapeutic agent against TNBCs and related cell lines is cisplatin (Figure 1A). Cisplatin has been widely used as antitumor agent in the clinics ever since its discovery in the 1960s. It is estimated that at least half of current therapy protocols employ platinum-based anticancer drugs [4]. Binding of platinum to DNA results in structural deformation of the double-stranded structure, which leads to inhibition of DNA replication and transcription. Subsequently, the DNA damage response leads to apoptosis. The toxicity of cisplatin to normal tissues, like neurotoxicity and hepatotoxicity, along with the acquired therapeutic resistance of cancer cells, reduce the clinical efficacy of this drug [5].

Curcumin, an extractive of turmeric root, is a natural polyphenol which has been widely reported to possess the anticancer properties (Figure 1B) [6]. The chemical groups of –OH and –OCH3 in curcumin structure, are reported to be responsible for the antioxidant and anti-proliferative properties, respectively [7]. However, the poor bioavailability of curcumin and its instability in physiological media have limited its therapeutic application in clinic [8]. The use of nano carriers in drug delivery systems may increase the solubility, reduce the required dose, attribute to the targeted delivery and, can prolong drug’s maintenance in the systemic circulation [9]. Recent works by our lab have shown that the nano-based compounds of curcumin could be employed as an anticancer agents in vitro and in vivo [10,11].

Combination therapies of natural compounds can be employed as a novel strategy in promoting routine drug efficiency and reducing side effects [12]. Curcumin increases the sensitivity of breast cancer cells to cisplatin through down-regulation of FEN1 [13]. It has been demonstrated that curcumin in combination with carboplatin induces apoptosis and suppresses metastasis in human lung and hepatic cancer cells [14,15]. Montopoli et al. suggested that curcumin is an interesting natural polyphenol capable of limiting cell proliferation and possibly, increasing clinical impact of platinum drugs in ovarian cancer patients [16]. More recently, a study confirmed the suppressive effect of curcumin on cisplatin resistance in colorectal cancer cells [17]. These findings support the auxiliary role of curcumin as an adjunct to current chemotherapies and indicate that curcumin could be more effective in combination with chemotherapeutic drugs. This phytochemical in the form of gemini curcumin is more advantageous because of its improved stability, cellular uptake and cytotoxicity (Figure 1C). Gemini surfactants are a class of nano-sized materials consisting of two identical structures liked by a spacer that are highly effective in delivering gene and drugs. Gemini-Cur can trigger apoptosis in cancer cells through modulation of cell cycle and up regulation of apoptotic genes [18].

Here, we investigated the therapeutic effect of the combination of Gemini-Cur and cisplatin as a novel potential therapy on resistance TNBC cells. Gemini-Cur reduces the resistance of MDA-MB-231 to cisplatin through induction of apoptosis.

## 2. Results

### 2.1. Gemini-Cur and Cisplatin Have a Synergistic Toxic Effect on MDA-MB-231 Cells

**Figure 2:**
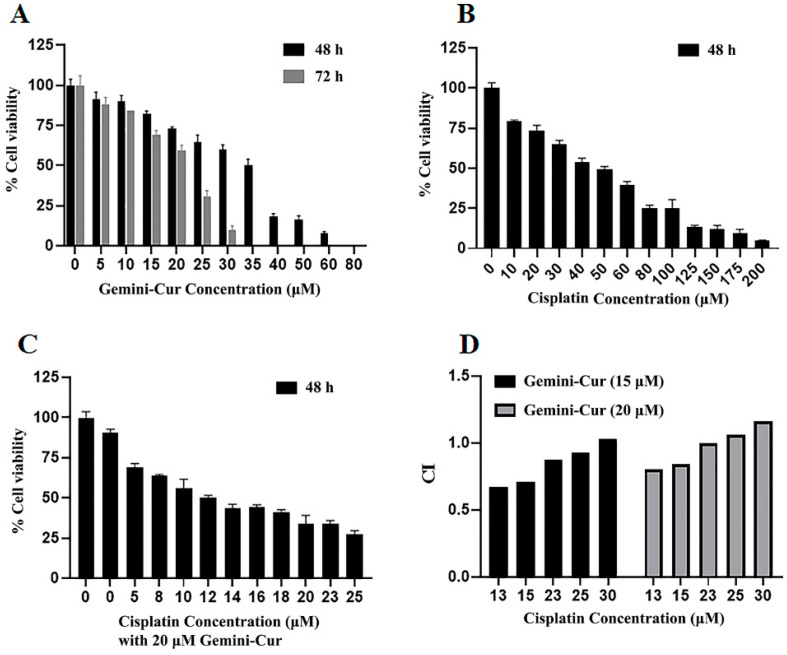
Effect of Gemini curumin and cisplatin on the viability of MDA-MB-231 cells in vitro. MDA-MB-231 cells were grown and treated with Gemini-Cur (**A**), Cisplatin (**B**) and Gemini-Cur/Cis (**C**,**D**). 15 and 20 μM of Gemini-Cur combined with serial concentrations of cisplatin. Data represent mean ± standard deviation of three independent experiments. CI: Combination index.

Breast cancer cells were treated with different concentrations of Gemini-Cur and cisplatin and the combination index (CI) was analyzed by using Chou–Talalay equation method [17]. Our results showed that Gemini-Cur has an inhibitory effect on the viability of MDA-MB-231 cells in a time- and dose-dependent manner with IC50 values of 35.06 and 23.48 μM in 48 and 72 h, respectively (Figure 2A). However, cisplatin lonely suppressed the proliferation of MDA-MB-231 cells with IC50 of 58.32 μM for 48 h. Then, serial doses of cisplatin and Gemini-Cur were employed and concentrations with CI < 1 were selected as proper ratio for further studies. The IC50 of cisplatin and Gemini-Cur was adjusted to 13 μM and 20 μM, respectively (Figure 2C). As shown in Figure 2D, the combination index is <1, pretending synergism between Gemini-Cur and cisplatin in 20 and 13 μM concentrations, respectively.

### 2.2. Morphological Visualization of Apoptosis

**Figure 3:**
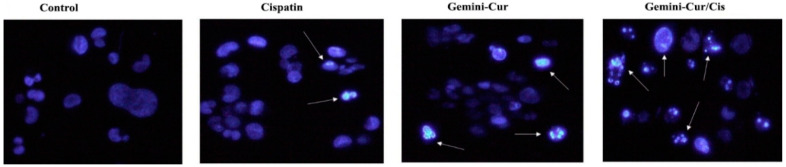
Morphological observation of apoptosis in Gemini-Cur and cisplatin treated cells by Hoechst staining in 48 h. Control shows a uniform exposure of live cells to stain. The color intensity and the number of cells indicating apoptotic features are significantly increased in treated groups. Arrows indicate apoptotic cells with cell shrinkage, nuclear fragmentation and DNA condensation. Magnification: 200×.

To visualize morphological changes in MDA-MB-231 cells, Hoechst staining was performed. As Figure 3 illustrates, TNBC cells undergo apoptosis after treatment with 20 μM Gemini-Cur and 13 μM cisplatin in both singular and combination forms. The microscopic visualization shows a uniformly light stain on the cells. However, a remarkable change in color intensity is clearly seen in treated cells with a significant difference in the number of apoptotic cells in Gemini-Cur/Cis treated group. The Hoechst staining clearly differentiate cells with nuclear fragmentation and DNA condensation as specified by arrows.

### 2.3. Gemini-Cur/Cis Modulates Cell Cycle Distribution in MDA-MB-231 Cells

**Figure 4:**
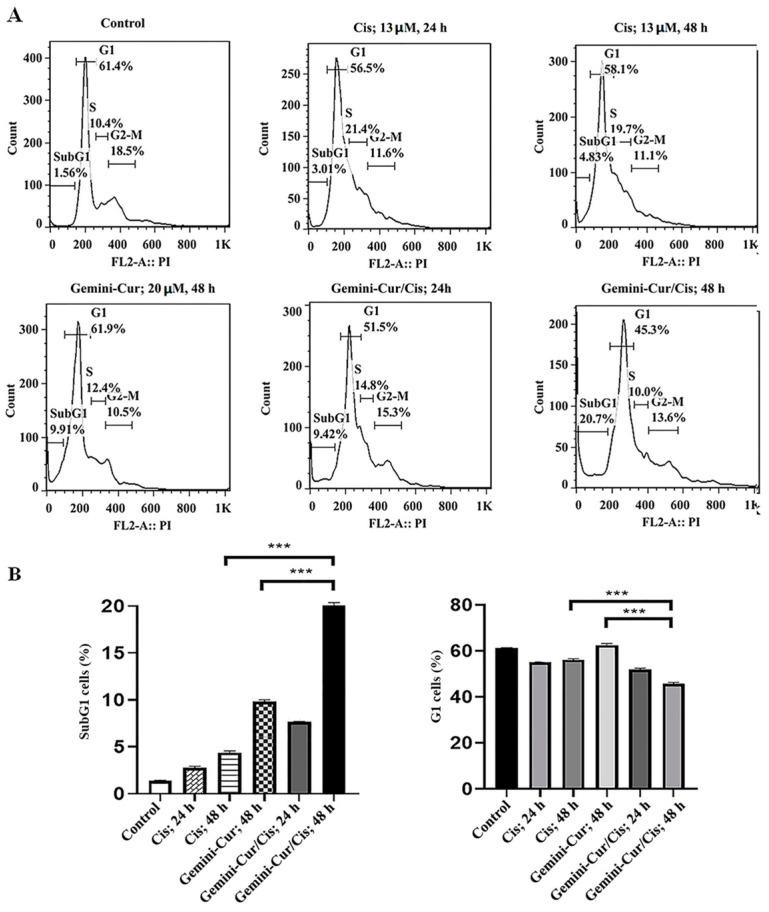
Analysis of cell cycle by flow cytometry in cancer cells treated with 13 μM cisplatin and 20 μM Gemini-Cur for 24 & 48 h. (**A**) Histograms for MDA-MB-231 cells. (**B**) The percentage of cells in sub-G1 and G1 phases. The data clearly illustrate an increase in the number of SubG1 cells in combination treatment compared to either void cisplatin or Gemini-Cur. Accordingly, the number of live cells is decrease in Gemini-Cur/Cis group versus Gemini-Cur and Cis groups. Data represent mean ± standard deviation of three independent experiments. (*** *p* < 0.001).

Cell cycle distribution was studied by propidium iodide staining in flow cytometry. Our data showed that cell cycle distribution is modulated in all treated cells either in singular or combined forms. As Figure 4 shows, the percentage of SubG1 cells as a hallmark of apoptosis is significantly increased in Gemini-Cur/Cis group compared to curcumin or cisplatin groups (*p* < 0.001). Accordingly, the number of live cells (G1 phase) was decrease in combination treatment when compared to the Gemini-Cue and Cis groups (*p* < 0.001). There was a statistically significant interaction between the effects of Cis and Gemini-Cur on interest in sub G1 (*p* = 0.0001).

### 2.4. Annexin V-FITC/PI Assay Confirmed Apoptosis in MDA-MB-231 Treated Cells

**Figure 5:**
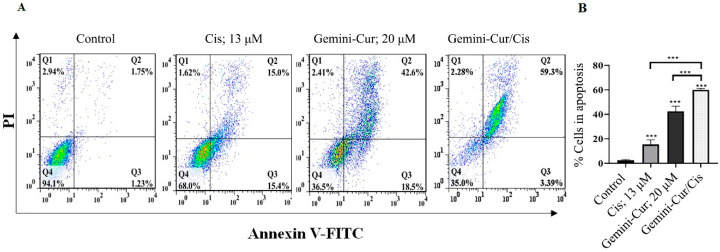
The combination of cisplatin and Gemini-Cur induces breast cancer cell apoptosis. MDA-MB-231 cells were treated with 13 μM cisplatin, 20 μM Gemini-Cur, and Gemini-Cur/Cis for 48 h. (**A**) plots show that live cells undergo apoptosis and the number of death cells is increased in treated cells, especially in Gemini-Cur/Cis group. (**B**) The proportion of apoptotic cells in late stage is significantly increased in combination treatment. ***: *p* < 0.001.

Annexin V-FITC/PI was employed to further confirm the mode of death in treated cells. As Figure 5 shows, apoptosis is induced in all treatments including 20 μM Gemini-Cur, 13 μM cisplatin and Gemini-Cur/Cis. However, the proportion of cells in late apoptosis is meaningfully increased to 59.3% in combination form compared to 15 and 42.6% in cisplatin and Gemini-Cur, respectively (*p* < 0.01).

### 2.5. Expression of BAX, BCL-2 Genes Are Modulated in Treated Cells

**Figure 6:**
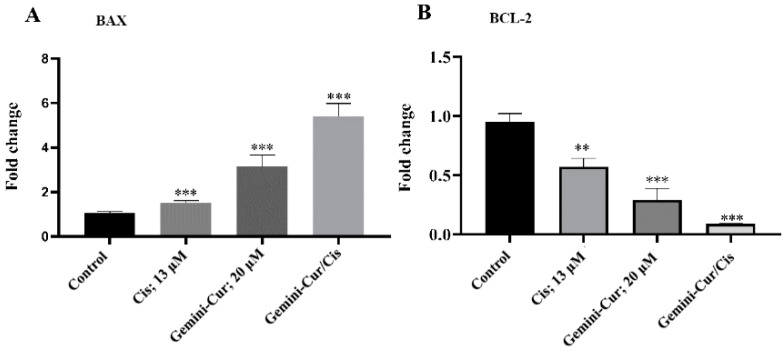
Quantitative analysis of the expression of apoptotic genes by real-time PCR. Relative expression for BAX (**A**) and BCL-2 (**B**) in MDA-MB-231 cells. Values represent mean ± standard deviation of three independent experiments; ** *p* < 0.01 and *** *p* < 0.001.

**Figure 7:**
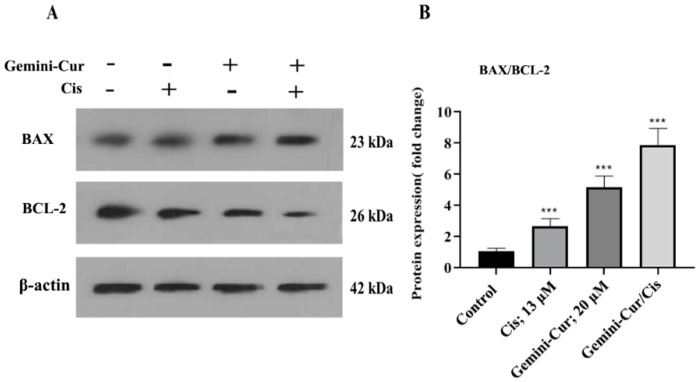
(**A**) Western blotting for BCL-2 and BAX (**A**) proteins. β-actin was used as internal control. (**B**) Data analysis showed that the protein ratio of BAX/BCL-2 is significantly increased in the cells treated with Gemini-Cur/Cis compared to the groups of Cis and Gemini-Cur. Values represent mean ± standard deviation of three independent experiments. *** *p* < 0.001.

The expression ratio of BAX/BCL-2 is usually considered as a hallmark of apoptosis. Real-time PCR demonstrated that BAX/BCL-2 expression is modulated in treated MDA-MB-231 cells. As Figure 6 shows, apoptotic BAX is upregulated while anti apoptotic BCL-2 is downregulated in all treatments. Furthermore, this modulation was significant in Gemini-Cur/Cis treatments rather than cisplatin or Gemini-Cur groups. This differential effect of combination treatment was clearly detected in protein level (Figure 7A). Further analysis demonstrated that the protein ratio of BAX/BCL-2 is significantly increased in combination treatments (*p* value < 0.01) and Gemini-Cur (*p* value < 0.001) groups.

## 3. Discussion

“Based on the St. Gallen/Vienna 2019 consensus discussion, 53% of the panelists didn’t accept the use of a platinum-based regimen in the neoadjuvant treatment of TNBC patients” [19]. Presently, there are limited criteria for TNBC treatment, and what could help medical oncologists to cure TNBC or prolong the survival rate in TNBC patients are novel therapeutic strategies [20]. The present study aimed to sensitize TNBC MDA-MB-231 cells to cisplatin through employment of novel curcumin nano formulation (Gemini-Cur).

Our data showed that cisplatin modulates the proliferation of MDA-MB-231 cells in high concentrations. However, its combination with appropriate dose of Gemini-Cur not only reduced the IC50 value of cisplatin to 13 μM but also, boosted its suppressive effect on the growth of MDA-MB-23 cells. Due to the nephrotoxicity and hepatoxicity of cisplatin, decreasing its effective dose in cancer treatments is of interest in clinic [4,5]. Different therapeutic compounds have already been employed to improve the toxicity of cisplatin. More recently, it has been reported that curcumin reduces the resistance of colorectal cancer cells to cisplatin [21]. Here, the employment of Gemini-Cur, has significantly reduced the effective dose of cisplatin to 13 μM, a concentration that may not indicate side effects. Cell cycle analysis and microscopic visualization also demonstrated that the mode of death induced in treated-TNBC cells is of apoptosis.

Since the growth inhibitory effect of Gemini-Cur/Cis was more effective than singular treatments, it seems that Gemini-Cur increases the sensitivity of MDA-MB-231 cells to cisplatin. The anticancer activities of natural compounds such as curcumin has been examined in combination with numerous chemotherapy drugs such as cisplatin. It was observed that curcumin affects the toxic properties of cisplatin in different cancer cells [12,13,15]. It has also been shown that curcumin inhibits the function of P-gp efflux pump in cancer cells that might support the reversal effect of this phytochemical on therapeutic resistance of cancer cells [22].

Here, we indicated that the expression ratio of BAX/BCL-2 is modulated in apoptotic cells. These proteins are both essential gateways to cell death. Numerous studies have revealed that curcumin triggers apoptosis through BAX/BCL-2 mediated pathway in cancer cells [8,14]. The data is also in concordance with results of Karimpour et al. for the modulatory effect of Gemini-Cur on the expression of apoptotic genes in breast cancer cell lines [18]. The up regulation of BAX protein and down regulation of BCL2 was clearly detected in Gemini-Cur/Cis group compared to controls.

Taken together, the current results illustrate the synergistic property of Gemini-Cur as a novel nano formulation of curcumin on the therapeutic potential of cisplatin against drug-resistant MDA-MB-231 cells. Enhancing the sensitivity of TNBC cells to cisplatin is still an interesting topic of cancer therapy for breast cancer. Therefore, it is worth investigating the exact cellular mechanisms and pathways involved in Gemini-Cur/Cis toxicity on TNCB cells. Furthermore, co-encapsulation of curcumin and cisplatin in gemini nanopartilces can be considered as an effective strategy to target cancer.

## 4. Materials and Methods

### 4.1. Reagents and Cell Culture

Curcumin and mPEG urethane gemini surfactant nanoparticles were a kind gift from Dr. Farhood Najafi at the Institute for Color Science and Technology, Tehran, Iran. Cisplatin was obtained from Mylan Corporation, Germany. Gemini curcumin was formulated by dissolving 6 mg of curcumin and 100 mg of gemini nanoparticles in 3 mL methanol by using sonication at room temperature. Then, the organic phase was evaporated by a rotary evaporator in room temperature. The encapsulated nano curcumin was dissolved in 10 mL distilled water and stored at 4 °C for further analyses [11].

Human breast adenocarcinoma cell line, MDA-MB-231, was obtained from the national cell bank of Iran (Pasteur Institute, Tehran, Iran). The cells were cultured in DMEM-High glucose medium (Sigma-Aldrich, St. Louis, MO, USA) supplemented with 100 U/mL penicillin, 100 mg/mL streptomycin, and 10% fetal bovine serum (Gibco, New York, NY, USA), and incubated at 37 °C in a humidified atmosphere of 5% CO_2_.

### 4.2. Cell Viability Assay

Cell viability was measured using the 3-[4,5-dimethylthiazol-2-yl] 2,5-iphenyltetrazolium bromide (MTT; Sigma-Aldrich, USA) assay. In brief, MDA-MB-231 cells were seeded into 96-well plates at a density of 10^4^ cells per well and cultured overnight. After 24 h, cells were treated with various concentrations of cisplatin (0–200 μM), Gemini-Cur (0–100 μM) and combinations of Gemini-Cur and cisplatin (Gemini-Cur/Cis) for 24 and 48 h at 37 °C. Then, 20 μL of 5 mg/mL MTT was added to each well and further incubated for 4 h. The medium was replaced with 150 μL of DMSO and thoroughly mixed. Optical absorbance value was recorded at 570 nm using a plate reader (Biotek, ELX808, Winooski, VT, USA). The IC50 values of cisplatin, Gemini-Cur, and Gemini-Cur/Cis were calculated using the Graphpad prism software version 8.0.2. (Graphpad Prism, La Jolla, CA, USA).

### 4.3. Analysis of Synergistic Cytotoxicity

The synergistic effects of cisplatin and Gemini-Cur were quantitatively analyzed by the calculation of the combination index (CI) using Compusyn software program, which utilizes the Chou–Talalay equation method [23]. CI analysis calculates the interaction between Gemini-Cur and cisplatin agents. The CI value was calculated using the following equation:

$$\mathrm{CI}=\frac{\mathrm{CA}.x}{\mathrm{ICx}.A}+\frac{\mathrm{CB}.x}{\mathrm{ICx}.B}$$

where CA.x and CB.x are the concentrations of agent A and agent B, respectively used in combination that inhibits cell growth by x%. The ICx.A and ICx.B are concentrations required for x% inhibition by agent A and agent B in singular form, respectively. Values of CI < 1, CI = 1, and CI > 1 indicate synergism, additive effect, and antagonism, respectively.

### 4.4. Visualization of Apoptosis by Hoechst Staining

MDA-MB-231 cells (4 × 10^5^) were seeded into 6-well plates for 24 h. Then, the cells were treated with 13 μM cisplatin and 20 μM Gemini-Cur, both in singular and combination forms. After 48 h, treated cells were collected and fixed with 3.7% paraformaldehyde for 30 min at room temperature, washed and stained with 167 μmol/L Hoechst 33258 at 37 °C for 30 min. Finally, cells were observed under a fluorescence microscope (RX50, LABEX, London, England) equipped with a UV filter.

### 4.5. Cell Cycle Analysis by Flow Cytometry

MDA-MB-231 cells (4 × 10^5^) were seeded into 6-well plates and treated with 13 μM cisplatin and 20 μM Gemini-Cur in singular and combination forms for 24 and 48 h. Treated cells were fixed using ice cold 70% ethanol, washed 2X with PBS and then resuspended with propidium iodide (10 mg/mL) and ribonuclease A (0.1%) for 30 min. Finally, the cells were incubated for 30 min in the dark place at room temperature. Fluorescent events from propidium iodide–DNA complexes were quantified by fluorescence-activated cell sorter (FACS) (BD Biosciences, Franklin Lakes, NJ, USA) with a count of 10,000 cells per sample. Finally, DNA contents at different phases of the cell cycle were determined by using FlowJo 7.6.1 Software.

### 4.6. Annexin V FITC/PI Assay

To further confirm apoptosis, annexin V/FITC assay (ApoFlowEx FITC Kit, Exbio, Czech RepubliC), was employed to detect the mode of death in MDA-MB-231 cells. Briefly, the cells were seeded on 6-well plates for 24 h. After 24 h, cisplatin (13 µM) and Gemini-Cur (20 µM) was added to different wells in both singular and combination forms. After 24 and 48 h, cells were collected, washed twice with PBS and suspended in 1oo µL binding buffer. Annexin V/FITC solution was added to the cells followed by addition of 10 µL PI. Stained cells were then detected with a flow cytometer (BD Biosciences, Franklin Lakes, NJ, USA).

### 4.7. Expression Studies by Real-Time PCR and Western Blotting

#### 4.7.1. Real-Time PCR

Total RNA was extracted with a selfmade TRIzol reagent, BRIzol, and followed by DNaseI treatment to eliminate any DNA content. The integrity and purity of RNA were evaluated using Pico200 (Picodrop, Hinxton, UK) and agarose gel electrophoresis. Total RNA was reverse transcribed into cDNA by employing PrimeScript RT Reagent Kit (Takara Bio, Kusatsu City, Japan). Real-time polymerase chain reaction (real-time PCR) was used to quantify the expression of BAX, BCL-2 and beta-2 microglobin (*β2M)*. Table 1 shows the characteristics of primers used in PCR. The comparative CT (2^−ΔΔCT^) method was used to determine the relative gene expressions [24].

#### 4.7.2. Western Blotting

To confirm the data of quantitative PCR, we evaluated gene expressions in protein level. Briefly, total protein was extracted from cells before and after treatment. Equal amounts of extracted proteins (40 mg/sample) were separated by SDS polyacrylamide gel electrophoresis and transferred to 0.45-mm polyvinylidene difluoride membrane. The membrane was blocked with 5% (*w/v*) non-fat dried milk (Difco/Becton Dickinson, Franklin Lakes, NJ, USA) and incubated for 2 h at room temperature with primary antibody [1:200, BCL-2 (sc-492), BAX (sc-7480), and β-actin (sc-47778)]. Mouse anti-rabbit IgG-HRP in 5% defatted dry milk-TBS-0.1% Tween (Santa Cruz Biotechnology, Dallas, TX, United States) were employed to visualize proteins. All steps were performed at room temperature. All signals were visualized using enhanced Western Blotting Luminol Reagent (Santa Cruz Inc., Dallas, TX, USA).

### 4.8. Statistical Analysis

All experiments were repeated at least three times and the data were expressed as mean ± standard deviation. SPSS 19.0 software was used in statistical analysis. Significance was determined using the one-way or two-way ANOVA test with a post hoc multiple comparison test. Meanwhile, the IC50 and combination index values were calculated using Graphpad prism version 8.0.2 and Compusyn software.

## 5. Conclusions

In conclusion, our data show that Gemini-Cur can improve the apoptotic effect of cisplatin on cancer resistant cells. Further studies are needed to figure out the molecular mechanism of synergistic effect of Gemini-Cur/Cis in TNCB cells.

## Figures and Tables

**Table 1:** Sequences of primers used in real-time PCR.

Genes	Primers	Size (bp)
β-actin	F: 5′-TGCCCATCTACGAGGGGTATG-3′ R: 5′-CTCCTTAATGTCACGCACGATTTC-3′	155
BAX	F: 5′-GCAAACTGGTGCTCAAGG-3′ R: 5′-ACTCCCGCCACAAAGA-3′	236
BCL-2	F: 5′-TGGGAAGTTTCAAATCAGC-3′ R: 5′-GCATTCTTGGACGAGGG-3′	298

## Data Availability

Data is contained within the article.

## Sample Availability

Samples of the compounds are available from the authors.
